# *Brassica napus* and diabetic complications

**Published:** 2015-10-03

**Authors:** Fatemeh Dehghan Shahreza

**Affiliations:** Department of Clinical Science, School of Veterinary Medicine, Ferdowsi University of Mashhad, Mashhad, Iran

**Keywords:** *Brassica napus*, Diabetes, Hyperglycemia, Hyperlipidemia, Antioxidant components

**Implication for health policy/practice/research/medical education:**

Hyperglycemia, hyperlipidemia and oxidative damage are major reasons of diabetes accelerated renal and cardiovascular diseases. Medicinal plants have attracted considerable attention as favorable curative applications in the treatment, alleviation and management of complications associated with chronic hyperglycemia and hyperlipidemia due to they contain various bioactive constituents and less toxic adverse effects, Brassica napus is one such plant.

## Introduction

Chronic hyperglycemia results in diabetes associated complications development that is nowadays a global health disorder without efficient therapeutic application. Hyperglycemia, hyperlipidemia and oxidative damage are major reasons of diabetes accelerated renal and cardiovascular diseases. Hyperglycemia enhances oxidative injury of cellular macromolecules by making disruption of ATP generation processes, activating polyol pathway, rising hexosamine process and formation of advanced glycation end products (AGEs). In fact, hyperglycemia induced oxidative stress increase micro-inflammatory pathways which lead to overexpression of adhesion molecules, enhancement of vascular permeability and infiltration of inflammatory cells into extracellular space. NADH oxidase enzyme contributes to reactive oxygenated species production in diabetic complications. Recent studies also have indicated that Nox4, an iso-enzyme of NADH oxidase family, is major source of superoxide radical and hydrogen peroxide in diabetic nephropathy (1).

Hyperlipidemia and diabetes associated complications are inseparably related together. In fact insulin resistance, alleviation of insulin production and release are responsible for alteration of normal lipid metabolism in adipose tissue. Thus, high level of free fatty acids is mobilized to liver. In addition, NADPH oxidase activity increases generation of radicals in adipose tissue that cause overproduction of pro-inflammatory adipocytokines, lead to systemic and local inflammation development (2).

Recently, medicinal plants have attracted considerable attention as favorable curative applications in the treatment, alleviation and management of complications associated with chronic hyperglycemia and hyperlipidemia due to they contain various bioactive constituents and less toxic adverse effects. *Brassica napus* is a flowering member of *Brassicaceae* family that is attributed to rape, rapeseed and oilseed rape. Mostly this plant is used for producing canola oil which is a good source of various chemoprotective agents. It has been determined consumption of vegetable and oilseed of brassica decrease age related chronic disease such as diabetes, renal failure, hypertension and atherosclerosis. Furthermore, it contains various polyphenolic antioxidants; anthocyanin, kaempferol and quercetin are major components. Other its bioactive constituents include carotenoids, vitamin C and E, folic acid, sinapic acid, lignans, glucose esters, phenolic acid, tripeptides. Sinapic acid is an effective peroxinitrit scavenger which inhibits modulation of necrotic or apoptotic pathways. Antioxidant value of brassica is affected by genotype and different parts of plant, environment and cooking methods. Brassica contributes in some biological actions such as antiviral, anti-carcinogenesis through repairing oxidative DNA damage, inhibitor of angiotensin convertor enzyme activity, reducer of serum low-density lipoprotein (LDL) level and enhancement of glucose tolerance (3). It is reported anthocyanin is potential component for reducing hyperglycemia associated complications through preventing AGEs formation. It is capable to capture methylglyoxal (MGO) and attenuate carbonyl groups (4).

In several studies has been examined antidiabetic property of brassica vegetable and oilseed in diabetic animal models.

In the study of Akbari and colleagues, on Alloxan induced diabetic rats, the impact of boiled extract of *Brassica napus* on hyperlipidemia and metabolic alterations were investigated. They concluded boiled extract of brassica significantly decreases cholesterol, LDL-C, triglyceride and glucose level and enhances high-density lipoprotein-*cholesterol* (HDL-C) in compared with control group (5).

Likewise, in the recent study that was conducted to examine hypoglycemic property of raw and cooked *Brassica napus* in Alloxan induced diabetic Wister rats. They divided 50 male Wistar rats into five groups which included control, diabetic control, diabetic raw *Brassica napus*, diabetic cooked *Brassica napus* and diabetic glibenclamide. They administered 3 g/BW cooked and raw *Brassica napus* extract to cooked Brassica diabetic rats and raw Brassica diabetic rats, respectively for 4 weeks. They observed the significant decrease of LDL-C, cholesterol, triglyceride and glucose serum level in raw and cooked brassica diabetic rats in compared control subjects. Also, both raw and cooked type of brassica administration led to enhancement of HDL-C. These results indicated raw and cooked *Brassica napus* extracts are able to improve serum level of lipid and glucose in diabetic rats. Interestingly, its cooked extract may be more promising therapeutic approach in diabetic patients (6).

Thus, *Brassica napus* has efficient antidiabetic and antioxidant components that may be a promising curative application for diabetic patient.

## Authors’ contribution

FDS is the single author of the paper.

## Conflicts of interest

The author declared no competing interests.

## Ethical considerations

Ethical issues (including plagiarism, data fabrication, double publication) have been completely observed by the author.

## Funding/Support

None.
